# Diagnostic Efficacy Study Comparing Tzanakis Scoring System With Alvarado Scoring System in Effective Diagnosis of Acute Appendicitis

**DOI:** 10.7759/cureus.58018

**Published:** 2024-04-11

**Authors:** Yathish Basava Prabhu BL, Bhupendra Mehra, Soumya Ghoshal, Siddharth P Dubhashi

**Affiliations:** 1 General Surgery, All India Institute of Medical Sciences, Nagpur, Nagpur, IND

**Keywords:** sensitivity and specificity, tzanakis score, alvarado score, negative appendicitis, appendicitis diagnosis

## Abstract

Objective

Among the common causes of abdominal emergencies, acute appendicitis ranks at the top, particularly in the young population. While negative appendectomy is not uncommon, the risk of appendicular perforation is substantial if the diagnosis is missed or delayed. This study evaluated the diagnostic efficacy of the Tzanakis scoring system for acute appendicitis, comparing it with the Alvarado scoring system, considering the histopathological finding as the gold standard.

Materials and methods

This prospective observational study, conducted in the General Surgery department in a tertiary care hospital in India, included clinically diagnosed acute appendicitis cases posted for open or laparoscopic appendicectomy.

Results

The mean age for the 60 participants included in the study was 30.97±13.44, and the median was 24.5 yrs. The sensitivity of ultrasonography (USG) in diagnosing histopathological positive acute appendicitis was 89%, and the specificity was 50%. The sensitivity, specificity, positive, and negative predictive values of the Tzanakis score were 87%, 50%, 96%, and 22%, respectively, and those of the Alvarado score were 54%, 75%, 96%, and 10%, respectively.

Conclusion

The receiver operator characteristic (ROC) curve for the Alvarado and Tzanakis scores showed that the area under the curve (AUC) was greater for the Tzanakis scoring system (0.670) than for the Alvarado scoring system (0.598). Differences between the AUCs were not statistically significant. Although the Tzanakis scoring system is more sensitive than the Alvarado scoring system in diagnosing acute appendicitis, studies with larger samples are needed to show the superiority of this scoring system over the Alvarado scoring system.

## Introduction

Inflammation of the appendix is one of the common causes of abdominal emergencies in the young population. It is more common in the younger age group than the adult population, as 40% occurs between 10 and 30 years of age. The lifetime risk of acute appendicitis is about 12% in males and 25% in females [1]. The diagnosis of acute appendicitis is mainly clinical. However, the diagnostic accuracy of clinical examination in acute appendicitis is approximately 89% [2]. The presentation may be atypical in about 18% of patients, which might lead to a false final diagnosis [3]. Whereas, if diagnosis is missed, acute appendicitis may lead to life-threatening complications. The median incidence of perforation is about 20%. Thus, the clinician needs to balance the risk of perforation with a negative appendicectomy. Ultrasonography (USG) is the primary radiological investigation for acute appendicitis. Though computed tomography (CT) scan is highly accurate, in most cases, it is utilised as a second-line investigation where the diagnosis is doubtful. Many scoring systems were developed to aid in diagnosing acute appendicitis, such as the Alvarado scoring system, Tzanakis scoring system, Raja Isteri Pengiran Anak Saleha (RIPASA) Score, and Appendicitis Inflammatory Response (AIR) score. The Tzanakis score and Alvarado scoring systems are frequently used throughout the Globe. A score ≥ 8 is required to diagnose acute appendicitis in the Tzanakis scoring system [4], and ≥7 is the cut-off for the Alvarado scoring system [5]. The reported sensitivity (Sn) and specificity (Sp) of the Tzanakis scoring system are 95.4% and 97.4 %, respectively [4]. The specificity and sensitivity of the Alvarado scoring system range from 87%-92% and 73%-90%, respectively [6,7]. The Tzanakis scoring system includes ultrasonography, clinical evaluation, and laboratory tests to assess acute appendicitis. The Alvarado and the modified Alvarado scoring systems mainly depend on clinical assessment and total leucocyte count (TLC) and do not include any radiological investigation.

Appendicectomy is a common emergency abdominal surgical procedure performed worldwide. A delay or misdiagnosis of appendicitis can result in severe complications such as perforation, abscess formation, sepsis, and intra-abdominal adhesions [8]. Our study aimed to assess the efficacy of the Tzanakis score and compare the Tzanakis and Alvarado scoring systems in diagnosing acute appendicitis and reducing the negative appendicectomy rate. Histopathological examination (HPE) was considered the gold standard.

## Materials and methods

**Figure 1:**
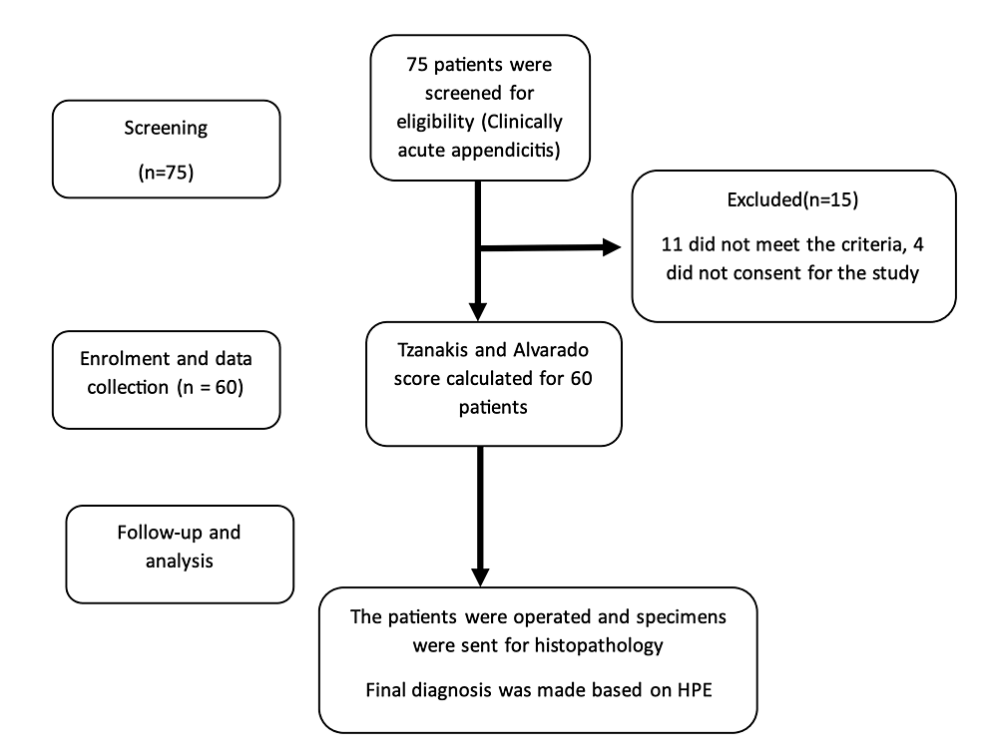
Study enrolment flow chart

This prospective observational study was conducted in the General Surgery department, AII India Institute of Medical Sciences, Nagpur, India, from July 2022 to January 2024. The Institutional Ethics Committee of All India Institute of Medical Sciences Nagpur (AIIMS Nagpur) approved the study (approval number: IEC/Pharmac/2022/428). The primary objective of this study was to determine the diagnostic efficacy of the Tzanakis scoring system in predicting acute appendicitis, considering the histopathological findings to be the gold standard. The secondary objective was to compare the effectiveness of the Tzanakis scoring system with the Modified Alvarado scoring system in predicting acute appendicitis. After the institutional ethical review board's approval, we enrolled patients in this study with their written informed consent. All the patients with clinically diagnosed acute appendicitis aged 18-60 years undergoing laparoscopic or open appendicectomy were screened for eligibility. We excluded patients aged less than 18 years and more than 60 years, patients not willing to participate, and patients with known psychiatric illness and appendicular mass formation. We assessed 75 patients for eligibility. Eleven patients did not meet the inclusion criteria. Four patients did not give consent. The study finally recruited 60 patients, clinically diagnosed to have acute appendicitis, who underwent emergency open/laparoscopic appendectomy (Figure 1).

Diagnosis of acute appendicitis and the decision of appendicectomy was made by the surgeon based on clinical examination findings aided by ultrasound examination. The Alvarado and Tzanakis scores (Table 1) were calculated at admission for all patients; however, scoring systems were not used to diagnose acute appendicitis. The cut-off score considered for the Tzanakis scoring system was ≥ 8, and that for the Alvarado scoring system was ≥7. A final diagnosis of acute appendicitis was made based on the HPE of the surgical specimen, which was considered the gold standard. The HPE features suggesting appendicitis include submucosa, muscularis propria, and serosa in the appendix, which show inflammatory infiltrates and associated peri-appendiceal fat stranding. Positive and negative appendicectomies were documented as per the histopathology report. The histopathological results were correlated with the patient's clinic-demographic characteristics Alvarado and Tzanakis scores. USG findings were compared between histopathological positive and negative appendicitis groups. Categorical variables were presented in number and percentage (%) and compared using the Chi-square test. Continuous variables were presented as mean ± standard deviation and median. Analysis was done with Statistical Package for Social Sciences (SPSS) version 26.0 (IBM Corp., Armonk, NY). The sensitivity, specificity, positive predictive value, negative predictive value, and diagnostic accuracy were derived at the optimal cut-off threshold for each scoring system. The receiver operator characteristic (ROC) curve was plotted. The diagnostic power of the two scoring systems was assessed by determining the area under the curve (AUC). The AUCs were compared with the gold standard under nonparametric assumption using Chi-square tests. A p-value of less than 0.05 was taken as significant.

**Table 1:** A summary of Alvarado, Tzanakis, Raja Isteri Pengiran Anak Saleha (RIPASA) Score, and Appendicitis Inflammatory Response (AIR) scoring systems and their components WBC - White blood cell, RLQ - Right lower quadrant, RIF - Right iliac fossa, USG - Ultrasonography

Scoring Systems	Components	Score
Alvarado Score	Migratory pain	1
	Anorexia	1
	Nausea or vomiting	1
	Tenderness in the right lower quadrant	2
	Rebound tenderness	1
	Elevation in temperature	1
	Leucocytosis (WBC count >10,000/mm³)	2
	Shift to the left in leukocytes	1
	Total Score Range	0-10
Tzanakis Score	Tenderness in the right lower quadrant	4
	Rebound tenderness	3
	Leucocytosis (WBC count >12,000/mm³)	2
	USG suggestive of acute appendicitis	6
	Total Score Range	0-15
RIPASA Score	Male	1
	Female	0.5
	Age <39.9 years	1
	Age >40 years	0.5
	Anorexia	1
	RIF pain	0.5
	Migration of RLQ pain	0.5
	Nausea and vomiting	1
	RIF tenderness	1
	RIF guarding	2
	Rebound tenderness	1
	Rovsing sign	2
	Fever	1
	Raised white cell count	1
	Negative urinalysis	1
	Total Score Range:	0-15
AIR Score	Right lower quadrant pain	1
	Vomiting	1
	Rebound tenderness	
	Light	1
	Medium	2
	Strong	3
	Fever > 38.5°C	1
	Leucocytosis (WBC count)	
	10,000-14,999/mm³	1
	>15000/mm^3^	2
	Neutrophil count	
	70-84%	1
	>85%	2
	C-reactive protein	
	10-49 mg/L	1
	>50 mg/L	2
	Total Score Range	0-12

## Results

For the 60 participants included in the study, the mean age was 30.97 ±13.44, and the median was 24.50 years. The age distribution in this study showed most cases in the age group 20-30 years. There were 40 male participants, 66.66%, and 20 female participants, the remaining 33.33% (N=60). The male-to-female ratio was 2:1. All patients had pain in the abdomen as the initial presenting feature. Nausea was one of the presenting symptoms in 46 patients (76.67%, N=60). Twenty-three patients (38.33%, N= 60) had a fever. Ultrasound examination revealed features of acute appendicitis in 52 patients out of 60 (86.67%). The appendix was found to be perforated in six patients(10%, N= 60). All patients underwent appendicectomy. Four patients out of 60 (6.67%) did not have findings of appendicitis on histopathological examination. Table 2 summarizes the clinicodemographic characteristics of the study population.

**Table 2:** Summary of clinicodemographic characteristics of the study population Data is presented as mean± SD for age and n (%) for other variables SD - Standard deviation, USG – Ultrasonography, HPE- Histopathology

Clinicodemographic features	Number (%)
Total patients	60 (100.00)
Male	40 (66.67%)
Female	20 (33.33%)
Mean age in years + SD	30.96 +13.44
Clinical findings	
Abdominal pain	60 (100.00)
Nausea	46 (76.67)
Fever	23 (38.33)
Anorexia	13 (21.67)
USG positive investigation	52 (86.67)
Total appendicectomy	60 (100.00)
HPE positive	56 (93.33)
HPE negative	4 (6.67)
Perforated appendix	6 (10.00)

The frequency distribution of clinicodemographic characteristics and imaging findings in histological positive and negative appendicitis cases are listed in Table 3. In most cases of appendicitis, the total leucocyte count (TLC) was below 10,000/mm. In 13 cases, the total leucocyte count was between 10,000-12,000/mm, suggesting mild inflammation. USG could detect evidence of appendicular inflammation in 83.3% (N= 60) of patients correctly. USG failed to detect appendicitis in six patients with histologically proven appendicitis (Table 3).

**Table 3:** Distribution of clinicodemographic characteristics and ultrasonography findings in histopathological positive and negative appendicitis Data is presented as mean± SD for age and n (%) for other variables SD - Standard deviation, USG - Ultrasonography

Clinicodemographic characteristics	Histopathology findings		
	Positive	Negative	Total (N=60)
Mean age (years)	31.20±13.50	27.80±11.40	30.96±13.44
Age groups, n (%)			
<20 years	12 (20.00)	1 (1.66)	13 (21.66)
20-30 years	24 (40.00)	2 (3.33)	26 (43.33)
30-40 years	5 (8.33)	0 (0.00)	5 (8.33)
40-50 years	7 (11.66)	1 (1.66)	8 (13.33)
50-60 years	8 (13.33)	0 (0.00)	8 (13.33)
Gender, n(%)			
Male	39(65.00)	1(1.66)	40 (66.66)
Female	17(28.33)	3(5.00)	20 (33.33)
USG findings, n(%)			
Negative	6(10.00)	2(3.33)	8 (13.33)
Positive	50(83.33)	2(3.33)	52 (86.6)
Total leucocyte count, n(%)			
<10,000 per microlitre	34(56.66)	2(3.33)	36 (60.00)
10,000-12,000 per microlitre	12(20.00)	1(1.66)	13 (21.66)
>12,000 per microlitre	10 (16.66)	1(1.66)	11 (18.33)
Clinical findings, n(%)			
Abdominal Pain	56(93.33)	4(6.66)	60 (100.00)
Nausea			
Present	43(71.66)	3(5.00)	46 (76.66)
Absent	13(21.66)	1(1.66)	14 (23.33)
Fever			
Present	22(36.66)	1(1.66)	23 (38.33)
Absent	34(56.66)	3(5.00)	37 (61.66)
Anorexia			
Present	12(20.00)	1(1.66)	13 (21.66)
Absent	44(73.33)	3(5.00)	47 (78.33)

Based on the Tzanakis scoring system, out of 60 patients (N= 60) who underwent appendectomy, 49 (81.6%) were found to have histopathology-proven acute appendicitis (true positive), and two (3.3%) were histopathology true negative (TN) ( Table 4). Acute appendicitis was significantly high (Odd's ratio 7, 95% Confidence interval 0.84-58.0, p-value = 0.042) in patients with a Tzanakis score of 8 or more.

**Table 4:** Distribution of Tzanakis score and histopathological examination result Data is represented as n (%) HPE - histopathological examination; TP - true positive; TN - true negative; FP - false positive; FN - false negative

Tzanakis Score	HPE positive	HPE negative	Total no of cases (N= 60)
≥ 8	49 (TP) (81.6%)	2 (FP) (3.3%)	51
< 8	7 (FN) (11.6%)	2 (TN) (3.3%)	9
Total	56	4	60

Based on the Alvarado scoring system, out of 60 patients (N=60) who underwent appendectomy, 30 (50%) were found to be true positive (HPE-proven acute appendicitis), and three (5%) were histopathology true negative (Table 5). Acute appendicitis was significantly high (Odd's ratio 3, 95% Confidence interval- 0.34 - 35.30, p-value = 0.042) in patients with an Alvarado score of 7 or more.

**Table 5:** Distribution of the Alvarado score and histopathological examination result Data is represented as n (%) HPE - histopathological examination; TP - true positive; TN - true negative; FP - false positive; FN - false negative.

Alvarado Score	HPE positive	HPE negative	Total number of cases (N= 60)
≥ 7	30 (TP) (50%)	1 (FP) (1.6%)	31
< 7	26 (FN) (43.3%)	3 (TN) (5%)	29
	56	4	60

The sensitivity of USG in diagnosing HPE-positive acute appendicitis was 89.1%, and the specificity was 50%. The sensitivity of the Tzanakis scoring system was 87%, and the specificity was 50%. The positive predictive value (PPV) was 96%, and the negative predictive value (NPV) was 22%. The Alvarado scoring system's sensitivity was 54%, and the specificity was 75%. The positive predictive value was 96%, and the negative predictive value was 10% (Table 6).

**Table 6:** Statistical analysis of Tzanakis score, Alvarado score, Ultrasonography Data is represented as % PPV - Positive predictive value, NPV - Negative predictive value, USG - Ultrasonography

	Tzanakis Score	Alvarado Score	USG
Sensitivity	87%	54%	89.1%
Specificity	50%	75%	50%
PPV	96%	96%	96%
NPV	22%	10%	25%
Diagnostic Accuracy	85%	55%	86.7%

**Figure 2:**
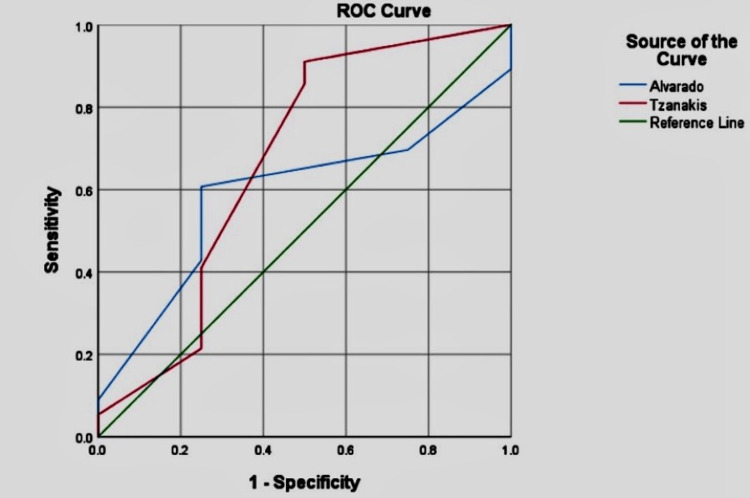
ROC curve for predicting acute appendicitis using Tzanakis and Alvarado score systems The area under the curve (AUC) for the Tzanakis scoring system was 0.670, and for the Alvarado scoring system was 0.589 ROC: Receiver operator characteristics curve

The receiver operator characteristics curve was plotted for the Alvarado and Tzanakis score. The area under the curve (AUC) for the Tzanakis scoring system was 0.670, with a standard deviation of 0.170. The AUC for the Alvarado scoring system was 0.589, with a standard deviation of 0.150 (Figure 2). When we compared both the tests with the gold standard examination (histopathology) by ROC curve plotting on the assumption of nonparametric distribution, there was no significant difference between the tests and the gold standard examination result (p-value = 0.260 and 0.553 for Tzanakis and Alvarado scores respectively).

## Discussion

Acute appendicitis is one of the most common causes of an acute abdomen. Multiple other emergency abdominal pathologies involving not only the right iliac fossa but also other regions of the abdomen may mimic acute appendicitis. Sometimes, it is challenging for the surgeon to make an accurate diagnosis. There is always a risk that the diagnosis is missed, leading to appendicular perforation, increasing morbidity and mortality. A negative appendectomy is also not unusual. A higher negative appendectomy rate of 15.0% to 25.0% was accepted formerly to avoid dreaded complications such as perforation and abscess formation [9]. Nevertheless, the removal of a normal appendix exposes the patient to all possible risks and complications of surgery and anaesthesia. The actual cause of abdominal pain remains undetected most of the time.

Most patients in this study were 21-30 years old. The male-to-female ratio is 2:1, which indicates male preponderance in the disease. Pain was the most common symptom, followed by nausea/vomiting and fever. The most common sign was pain in the abdomen, seen in all 60 cases in this study. In most cases of appendicitis, the TLC was well below 10,000/mm. In 13 cases, the counts were between 10-12,000/mm, suggesting mild inflammation. The sensitivity of USG in diagnosing HPE-positive acute appendicitis was found to be 89.1%, and the specificity was 50%. CT abdomen was done in 11 cases, and all were positive for the features of an inflamed appendix, further reaffirming that it is a better imaging modality than ultrasonography as it is operator-independent.

Even with the advancement in the field of modern diagnostic technologies, surgeons sometimes find it challenging to diagnose acute appendicitis correctly [10-12]. As the USG findings are operator-dependent, the sensitivity and specificity of USG in diagnosing acute appendicitis varies across different studies [12]. A contrast-enhanced CT scan of the abdomen is highly sensitive and specific when making a diagnosis. CT scan has sensitivity and specificity of 95% and 93%, respectively [13]. However, imaging modalities like CT and MRI are not readily available at many centres and are costly, which keeps them out of reach for most patients [14]. Also, a CT scan has its limitations owing to its ionizing radiation.

Numerous scoring systems have been proposed to support the diagnosis of acute appendicitis (Table 1). Most are complex and challenging to implement in a clinical setting [15]. Alvarado scoring system is the most widely used worldwide [16]. It is simple, easy to use and can successfully predict acute appendicitis [17]. The reported sensitivity and specificity of the Alvarado scoring system varies in the literature. Awayshih et al. reported the sensitivity and specificity of the Alvarado score to be 54% and 75%, respectively [18]. In contrast, another study comparing the accuracy of the Alvarado score with the RIPASA (Raja Isteri Pengiran Anak Saleha Appendicitis) score found these values to be 71.1% and 75.8%, respectively [19]. Overall, the sensitivity of the Alvarado score is consistently low throughout the literature. In the present study, based on the Alvarado scoring system, out of 60 patients who underwent appendectomy, 30 patients were found to be true positive, and three were true negative. The sensitivity was 54% and specificity 75%. The positive predictive value (PPV) is 96%, and the negative predictive value is 10%. Precise history and clinical examination are the two main components of the Alvarado scoring system. Due to its reliance on only signs and symptoms and not on imaging modalities, the sensitivity and specificity may be low. Moreover, different practitioners may have different approaches to getting correct patient information and interpreting clinical findings, which may contribute to reducing the sensitivity. In contrast, the Tzanakis score utilises a USG scan and clinical findings. It is expected to be more effective than the Alvarado scoring system. The present study was undertaken to test the efficacy of the Tzanakis score in preventing negative appendicectomy rates.

Based on the Tzanakis scoring system, out of 60 patients who underwent appendectomy, 49 were found to be true positive, and two were true negative. The sensitivity was 87% and specificity 50%. The positive predictive value (PPV) was 96%, and negative predictive value was 22%. The sensitivity, positive predictive value, and diagnostic accuracy of the Tzanakis score were comparable with those reported in the original study by Tzanakis et al. [4]. However, specificity was lower than that reported in the original research describing the development of the Tzanakis system [4]. The low specificity of the Tzanakis score may be due to our study's lower sensitivity rate of USG. Experienced radiologists and newer advanced USG technology can improve USG sensitivity. Ultrasound examination is operator-dependent and has variable sensitivity and specificity levels, 75-90% and 86- 100%, respectively [20].

The area below the curve in the receiver operator characteristics curve was more for the Tzanakis scoring system (0.670) compared to the Alvarado scoring system (0.598), which indicates that when diagnosing acute appendicitis, the Tzanakis scoring system is more accurate than the Alvarado scoring system. We found no significant differences when we compared the area under the curve for each scoring system with the gold standard under the assumption of nonparametric distribution.

Though the difference between the accuracy of Tzanakis and Alvarado scoring systems was not significant, the result of our study agrees with other published literature. The magnitude of the difference in the accuracy of the scoring systems is variable in different studies; most of them demonstrated that the Tzanakis is the better system of the two [20]. This difference may be due to the observer dependency in USG scans. In low-resource countries, where the USG scan is being increasingly used in different setups, the Tzanakis scoring system may be helpful for correctly diagnosing acute appendicitis. It also lowers the negative appendicectomy rate. Investigations like contrast-enhanced CT scans and diagnostic laparoscopy are usually expensive and may not be available in resource-restricted settings. However, searching for a more appropriate scoring system should always be desired.

Scoring systems are primarily helpful in suspected cases of acute appendicitis. The main limitation of this study was that it targeted patients who had already been clinically diagnosed with acute appendicitis and planned for operation rather than those patients who were suspected of having appendicitis. Also, this study had a relatively small sample size. For more explicit results, we need a larger sample size to prove the superiority of one scoring system to another. Another drawback of this study is that the various pathologists reported the histological examination of the specimens, where opinions might differ from person to person. Moreover, different surgeons and radiologists performed clinical and USG evaluations, possibly leading to inter-observer variations.

## Conclusions

Even though we have reasonably reliable scoring systems, most depend solely on signs and symptoms and do not include imaging techniques. Fortunately, the USG scan has now become more accessible, even in developing countries, and it can significantly help the surgeon make an accurate diagnosis. Even though there was no marked difference between Tzanakis and Alvarado scores in our study, it is essential to note that the Tzanakis scoring system has always had an edge over conventional scoring systems, no matter how small or large. Thus, scoring systems can help surgeons avoid advanced investigations like CT scans for diagnosis and management, which may be necessary in cost-sensitive developing countries.
